# Entomopathogenic fungi: beyond biocontrol-unravelling mechanisms of enhanced plant abiotic stress tolerance

**DOI:** 10.1007/s44297-026-00077-4

**Published:** 2026-05-11

**Authors:** Ya-Qiang Zheng, Komivi Senyo Akutse, Song Mei, Artemio Mendoza-Mendoza, Bin Chen

**Affiliations:** 1 College of Pharmacy, Guizhou University of Traditional Chinese Medicine, Guizhou Province, Guiyang, China; 2 International Centre of Insect Physiology and Ecology (icipe), P.O. Box 30772-00100, Nairobi, Kenya; 3 Unit of Environmental Sciences and Management, North-West University, Potchefstroom, 2520 South Africa; 4 Wine, Food and Molecular Biosciences Department, Lincoln University, Lincoln, Canterbury, 7674 New Zealand; 5 State Key Laboratory of Conservation and Utilization of Biological Resources of Yunnan, College of Plant Protection, Yunnan Agricultural University, Kunming, China

**Keywords:** Entomopathogenic fungi, Fungal endophytes, Abiotic stress, Drought tolerance, Salinity, Heavy metals, Plant–microbe interaction, Sustainable agriculture

## Abstract

Rising global food demand requires sustainable strategies to mitigate crop yield losses caused by biotic and abiotic stresses. Entomopathogenic fungi (EPF), traditionally used as biocontrol agents against insect pests, also exhibit a remarkable ability to live within plants as endophytes, significantly boosting plant resilience to abiotic stresses. This review summarises the mechanisms by which EPF endophytes colonise plants and confer robust tolerance to drought, salinity, and heavy metal toxicity. These beneficial fungi orchestrate host physiological adaptations by activating antioxidant systems, up-regulating stress-responsive genes, modulating phytohormone signalling, and enhancing nutrient assimilation. Through improved photosynthetic efficiency, osmotic adjustment, nutrient uptake, and ion homeostasis, EPF endophytes substantially increase plant biomass, yield stability, and productivity under severe stress. Harnessing these fungi as dual-purpose bioagents offers a sustainable alternative to chemical pesticides, bridging ecological pest and disease management and climate-resilient agriculture. Future efforts must prioritise optimising field efficacy and overcoming regulations and commercialisation barriers to unlock their full potential in sustainable global food systems.

## Introduction

Entomopathogenic fungi (EPF) are a diverse group of parasitic microorganisms that have evolved to infect and kill arthropods, including major members from the phyla Microsporidia, Chytridiomycota, Entomophthoromycota, Basidiomycota, and Ascomycota [1]. There are over a thousand known fungal species capable of killing insects, most of which belong to the ascomycete group. Species such as *Metarhizium anisopliae*, *M. robertsii*, *M. acridum*, and *Beauveria bassiana* have been developed as eco-friendly biological control agents against various insect pests [2]. These fungi affect 20 of 31 insect orders [1] and are found in nearly all terrestrial ecosystems, where they play a vital role in regulating insect populations [3, 4]. EPF constitute a diverse, systematised, heterogeneous group that varies in their biology. The majority of EPF are pathogenic to insects, demonstrating a high degree of effectiveness in infecting their hosts, which can act as regulators to minimise populations of harmful insects. EPF are phylogenetically, morphologically, and ecologically distinct from one another. The most widely used entomopathogenic strains belong to the genera *Beauveria* spp., *Metarhizium* spp., *Hirsutella* spp., *Lecanicillium* spp., *Verticillium* spp., *Isaria* spp., and *Paecilomyces* spp. EPF controls a wide range of insect species across their different stages, from larvae to adult hosts. All developmental stages of insects, including eggs, larvae, pupae, nymphs, and adults, can be infected by EPF. Their mode of action and virulence notably differentiate EPF. The degree of attachment and the ability of fungi to penetrate the host exoskeleton determine the infection success rate and the extent of infection [5].

Field application of EPF agents primarily relies on foliar spraying, with inoculum adhering to and breaching the insect cuticle before proliferating within the host hemocoel, ultimately leading to epizootics and a subsequent decline in pest populations [5–7]. Nevertheless, the practical efficacy of these EPF agents is frequently constrained by abiotic stressors prevalent in agroecosystems. Key impediments include ultraviolet (UV) radiation, ambient temperature extremes, and suboptimal relative humidity, all of which can compromise conidial longevity and virulence [8, 9]. Specifically, UV exposure and thermal stress are principal determinants of virulence, conidial depletion and field persistence [10, 11]. Appropriate mass-production and formulation strategies for EPF could partially solve these challenges. However, the most fundamental strategy to guarantee high virulence and infectivity is the selection of environmentally competent fungal strains that can persist in the host environment for the required infection period [10].

**Fig. 1:**
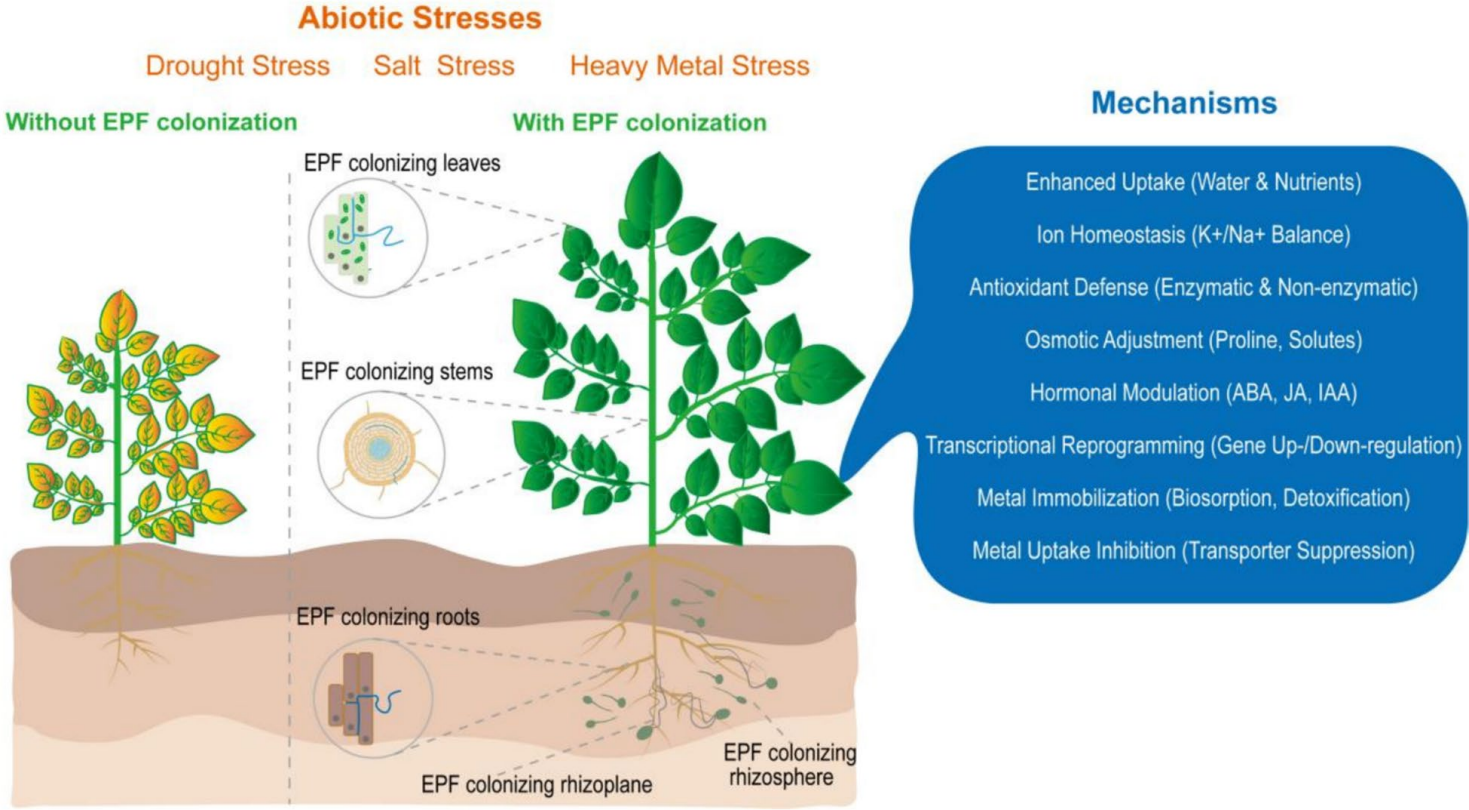
Mechanisms of abiotic stress mitigation in plants by entomopathogenic fungi. EPF: entomopathogenic fungi; ABA: abscisic acid; JA: jasmonic acid; IAA: indole-3-acetic acid. The figure was created with Adobe Illustrator

In recent years, EPF have also been found to act as endophytes by colonizing various host plants (Fig. 1) [12–15], so they are collectively referred to as endophytic insect‐pathogenic fungi (EIPF) [16]. Colonized fungi may not only directly infect herbivorous pests but also indirectly enhance plant defense against pests [17–20]. Furthermore, endophytic EPF can also promote plant growth and development [15, 21, 22].

The mechanisms that enable EPF to function as endophytes are still being unraveled. In recent years, evidence has suggested that *Metarhizium* and *Beauveria* species are closely related to endophytes and/or plant pathogenic fungi, such as *Fusarium* spp., associated with the Arabidopsis root mycobiome [23–25]. The evolutionary histories of *Metarhizium*’s entomopathogenicity and endophytism may differ from those of *Beauveria* spp. and other fungi [26]. The *Metarhizium* lineage, for instance, may have evolved from saprophytes that were first attracted to plant root exudates, became endophytes, and later acquired the ability to infect insects [22, 27]. Many *Metarhizium* species retain this flexibility, living as saprobes, insect pathogens, and plant symbionts simultaneously, with the ability to switch between these roles [22, 28]. This multifunctionality makes endophytic EPF a promising, eco-friendly alternative to chemical pesticides in Integrated Pest Management programs [13].

EPF play a significant role in alleviating both biotic and abiotic stresses in plants as endophytes. While previous reviews have comprehensively addressed the role of EPF as biocontrol agents against biotic stresses [13, 29–34] and others have broadly discussed fungal endophytes in stress tolerance [35], a critical gap remains in the systematic synthesis of EPF-specific mechanisms in abiotic stress alleviation. This review provides the first comprehensive synthesis that (i) integrates the dual roles of EPF endophytes in combating drought, salinity, and heavy metal stress within a unified framework and (ii) comparatively analyzes the mechanistic pathways (antioxidant, hormonal, nutritional) across different stress types (Fig. 1).

### Role of entomopathogenic fungi endophytes in mitigating abiotic stresses

Abiotic stresses, including drought, salinity, heat, and heavy metal toxicity, are significant environmental challenges that profoundly impact agricultural productivity, contributing to global yield losses exceeding 50% [35]. These stresses cause irreversible damage, leading to stunted growth, physiological injuries, and even plant death. They also inhibit growth, disrupt metabolic processes, and ultimately reduce both biomass accumulation and active component concentrations within plants [36–38]. A substantial body of evidence demonstrates that fungal endophytes play a central role in enhancing plant resilience to these detrimental abiotic stresses.

### Drought stress

Drought stress is the most common and widespread environmental factor that severely restricts crop productivity [39]. Global climate change is increasing the frequency of severe and extreme drought, making this challenge worse [40]. Drought-caused loss in crop yield probably surpasses losses from all other factors, since both the intensity and length of the stress are crucial factors of the final effect [41]. Emerging evidence [42, 43] demonstrates that EPF, particularly *Beauveria* and *Metarhizium* species, can alleviate drought stress through coordinated physiological, biochemical, and molecular mechanisms (Table 1).

**Table 1:** Mechanisms of entomopathogenic fungi (EPF) in alleviating plant drought stress

Mechanism	Key Actions	Observed Effects & Supporting Evidence
Water conservation, uptake, and nutrient mobilization	Maintains leaf water status and stomatal conductance	Higher leaf RWC and stomatal conductance in red oak seedlings colonized by *B.* *bassiana* [44]
	Promotes root system development	Enhanced root growth, critically improving water uptake and plant survival under severe drought in red oak [44]
	Improves plant acquisition of soil minerals	Enhanced uptake of P, Ca, Mg, and Fe in onion, supporting growth and secondary metabolite synthesis under drought (*B.* *bassiana*) [45]
Antioxidant Defense Activation	Boosts the activity of antioxidant enzymes	Increased activity of POD in tomato, and SOD, CAT, and APX in wheat and onion, leading to reduced oxidative damage (MDA, H₂O₂). Evidence from *B.* *bassiana* in tomato and onion [45, 46] and *M.* *anisopliae* in wheat [47]
	Induces synthesis of non-enzymatic antioxidants	Induced accumulation of non-enzymatic antioxidants (polyphenols, flavonols, ascorbic acid) that scavenge reactive oxygen species, thereby mitigating oxidative DNA damage and preserving genome stability under drought conditions [45, 48]
Osmotic Regulation	Modulates osmolyte concentration in plant cells	Increased proline content in tomato (*B.* *bassiana*) [44] and decreased proline in red oak [44] (*B.* *bassiana*), indicating species-specific strategies to maintain cell turgor
Phytohormone Modulation Genetic Regulation	Alters endogenous hormone levels	Increased gGA levels in *M.* *parviflora* primed with *B.* *bassiana*, promoting growth under drought stress [48]
	Upregulates host gene expression	Upregulation of key genes related to stomatal regulation and drought tolerance in tomato plants colonised by *B.* *bassiana* [46]

#### Water conservation, uptake, and nutrient mobilization

Endophytic EPF colonization enhances plant drought tolerance by improving water retention, promoting water uptake, and boosting nutrient acquisition. These interconnected strategies help plants maintain physiological function when water is limited.

*Water conservation and uptake*. EPF help plants manage water through multiple routes. In red oak seedlings, *B. bassiana* colonization maintained higher leaf relative water content (RWC) and stomatal conductance under severe drought while also promoting root growth to improve water access [44]. In *Malva parviflora*, *B. bassiana* priming enhanced leaf water status and reduced yield losses under water-limited conditions [48]. These improvements can translate into different water-use strategies depending on the plant-fungus combination. In tomato, for instance, *B. bassiana* colonization promoted a "water spender" strategy, and plants grew more and absorbed more water, supported by better root function and stomatal regulation [46].

*Nutrient mobilization.* Beyond water, EPF help plants acquire essential nutrients that support stress tolerance. In onion, *B. bassiana* colonization enhanced the uptake of phosphorus (P), calcium (Ca), magnesium (Mg), and iron (Fe) under drought [45]. These nutrients play key roles in energy transfer, membrane stability, chlorophyll synthesis, and antioxidant defense. In *M. parviflora*, priming with *B. bassiana* increased potassium (K) and magnesium (Mg) levels in both shoots and roots, contributing to better osmotic balance and photosynthetic function [48]. The fungus likely aids nutrient acquisition by extending the reach of roots, solubilizing minerals in the soil, or enhancing the expression of nutrient transporters.

Together, these findings show that EPF support plants under drought not only by helping them hold onto water but also by improving their ability to take up water and nutrients from the soil. This dual role, enhancing both water relations and nutritional status, helps explain the broad benefits of EPF colonization observed across different crop species.

#### Activation of antioxidant defense systems

Drought stress induces reactive oxygen species (ROS) accumulation, causing oxidative damage. EPF endophytes counteract this by boosting both enzymatic and non-enzymatic antioxidant systems. At the enzymatic level, EPF colonization boosts the activity of key ROS-scavenging enzymes. For example, *B. bassiana* increased peroxidase (POD) activity in tomato [46] and enhanced superoxide dismutase (SOD), catalase (CAT), and ascorbate peroxidase (APX) in onion [45]. In wheat, seed priming with *M. anisopliae* MetA1 significantly reduced lipid peroxidation and superoxide radical generation through the coordinated upregulation of SOD, CAT, and APX [49]. Similarly, *B. bassiana*-primed *M. parviflora* showed increased CAT, POD, and total antioxidant capacity (TAC), which correlated with lower levels of oxidative stress markers [48]. Across multiple plant species, this enzymatic activation consistently leads to reduced malondialdehyde (MDA) accumulation and improved membrane stability [45–49].

In parallel, EPF stimulate non-enzymatic antioxidant systems by promoting the accumulation of secondary metabolites with ROS-scavenging properties. In onion, *B. bassiana* inoculation elevated total polyphenol and flavonoid contents, as well as overall antioxidant capacity, effects that were most pronounced under severe drought, underscoring the critical role of fungal endophytes during acute stress [45]. In *M. parviflora*, priming with *B. bassiana* enhanced the levels of total phenolics (TPC), total flavonoids (TFC), and ascorbic acid (AA). These changes were accompanied by reduced oxidative damage (lipid peroxidation, H_2_O_2_, ROS) and improved genome stability [48].

Together, the coordinated enhancement of both enzymatic and non-enzymatic antioxidants reflects a multilayered defense strategy through which EPF help plants mitigate oxidative stress and maintain physiological function under drought conditions.

#### Modifying plant osmotic properties

Osmolyte accumulation is a central drought adaptation mechanism, but its regulation by EPF shows species-specific patterns. Proline accumulation is a common response to EPF colonization under drought. *B. bassiana* increased proline content in tomato [46] and onion [45], improving their ability to retain water and maintain cell pressure. Similarly, wheat seeds primed with *M. anisopliae* MetA1 showed higher proline levels under both moderate and severe drought [47].

EPF also promotes the accumulation of other osmolytes. In *M. parviflora*, *B. bassiana* priming elevated soluble sugars and free amino acids, which helped maintain osmotic balance and protect cell membranes [48]. However, this response is not universal. In red oak seedlings, *B. bassiana* conferred drought tolerance without increasing proline; in fact, proline levels were lower in colonized plants than in controls [48]. This suggests that some plants rely on alternative strategies, such as enhanced root growth, to cope with water stress, highlighting the context-dependent nature of EPF-mediated osmotic adjustment. Overall, modulating compatible solutes is an important part of how EPF help plants withstand drought, but its contribution varies across different plant-fungus combinations.

#### Hormonal and molecular regulation

Plants rely on a network of hormones to coordinate their response to drought stress. EPF can influence this network, shifting the hormonal balance in ways that help plants cope with water deficit. Abscisic acid (ABA) is a central player in the drought response, triggering stomatal closure to reduce water loss. In tomato, *B. bassiana* colonization upregulated genes involved in stomatal regulation and ABA signaling, pointing to improved control of water use at the molecular level [46]. In maize, plants colonized by *M. robertsii* showed elevated ABA levels under drought, alongside changes in related hormones such as jasmonic acid [50]. These adjustments help plants fine-tune their response to limited water. Gibberellins (GAs) promote growth and are often suppressed under stress. However, EPF can help maintain GA levels during drought. In *M. parviflora*, *B. bassiana* priming increased GA content while reducing ethylene, a hormone linked to stress-induced senescence [48]. This shift supports continued growth and delays aging, giving plants a better chance to recover when water returns. Other hormones also come into play. In maize, *M. robertsii* colonization altered the levels of jasmonic acid (JA) and its derivatives in ways that depended on both water availability and the presence of the fungus [50]. This suggests that EPF help plants balance growth and defense depending on the circumstances. In *M. parviflora*, the reduction in ethylene further contributed to stress relief, as high ethylene levels can accelerate tissue damage under drought [48].

At the molecular level, these hormonal shifts are accompanied by changes in gene expression. In tomato, plants colonized by *B. bassiana* showed upregulation of key genes related to stomatal regulation and drought tolerance, providing a direct link between fungal presence and enhanced stress adaptation [46]. While significant progress has been made in understanding hormonal and molecular regulation, the precise molecular dialogue between EPF and their host plants under drought remains largely unexplored. Deciphering this conversation is crucial for understanding why outcomes vary and for harnessing these interactions effectively.

Collectively, the evidence presented above demonstrates that EPF can be effective and versatile allies in enhancing plant drought tolerance through a suite of coordinated mechanisms. However, the outcomes of EPF inoculation are not universally positive. Peterson et al. [50] found that *M. robertsii* root colonization positively correlated with maize height under drought, suggesting growth-promoting potential. In contrast, Ahmad et al. [51] reported no significant drought tolerance enhancement in maize using the same fungus, likely due to differences in inoculation methods, and seed inoculation proved more effective than soil drenching [50, 51]. It is well known that inoculation methods greatly affect how well EPF colonize plants, and the optimal method often varies by fungal species or even strain [6]. In other words, how we apply these fungi can determine whether they establish successfully and, ultimately, whether they help plants cope with stress. Therefore, optimizing inoculation strategies is essential for both research and practical applications.

### Salt stress

Salt stress significantly affects crop growth and productivity, particularly in arid climates. Over 800 million hectares of soil worldwide are affected by salinity, with NaCl-induced salinisation posing the greatest threat to plants [52–54]. This soil condition disrupts physiological processes and osmotic and ionic balance and leads to oxidative stress, which can impair photosynthesis and reduce crop yields [55–58]. To cope with salt stress, plants have evolved complex physiological, biochemical, and molecular mechanisms, including ion homeostasis, osmotic adjustment, antioxidant defense, and hormone modulation [56–58]. Emerging evidence indicates that EPF endophytes can enhance plant salt tolerance through multiple coordinated mechanisms (Table 2).

**Table 2:** Systematic summary of EPF-mediated mechanisms in alleviating salt stress

Mechanism	Key Actions	Observed Effects & Supporting Evidence
Ion Homeostasis	Maintain or enhance K⁺/Na⁺ ratio; reduce sodium ion accumulation	Improved K⁺/Na⁺ ratio in rice under salt stress [59]; reduced Na⁺ toxicity in rice [60]
Osmotic Adjustment	Promote accumulation of compatible solutes (proline, carbohydrates)	Increased proline content in soybean and rice [59–61]; enhanced osmolyte accumulation in rice [60]
Antioxidant Defense Activation	Boost enzymatic (CAT, POD, APX, SOD) and non-enzymatic (phenolics, flavonoids) antioxidant systems	Reduced oxidative damage (lower ROS, MDA) in rice [59]; activated antioxidant enzymes in rice and potato [60–62]; produced antioxidant compounds in tomato [63]
Phytohormone Modulation	Modulate hormone balance: decrease ABA; increase JA; maintain IAA production; modulate ethylene via ACC deaminase	Decreased ABA and increased JA in soybean [61]; sustained IAA production and ACC deaminase activity under high salinity [63]
Direct Growth Promotion	Directly stimulate growth under saline conditions via integrated mechanisms	Improved shoot length, biomass, chlorophyll content, leaf area, leaf succulence, RWC, and yield in soybean, rice, potato, and tomato under salt stress [59–63]

#### Ion homeostasis 

A central mechanism of salt tolerance is the maintenance of intracellular ion balance, particularly the K^**+**^/Na^**+**^ ratio. EPF colonization has been shown to improve this ratio in several crops. In rice, seed priming with *M. anisopliae* MetA1 significantly lowered Na^**+**^ accumulation and increased K^**+**^ content in both shoots and roots, leading to a reduced Na^**+**^/K^**+**^ ratio under salt stress [59]. Similarly, *B. bassiana* BeauA1 improved the K^**+**^/Na^**+**^ ratio in rice under both saline and non-saline conditions [60]. These effects likely involve the regulation of ion transporters, although the molecular players, whether fungal-derived signals or plant-induced transporters, remain to be identified.

#### Osmotic adjustment through compatible solute accumulation

The accumulation of osmolytes such as proline and soluble carbohydrates is a common response to salt stress, helping to maintain cell turgor and protect cellular structures. EPF endophytes promote this adaptive response. In soybean, *M. anisopliae* LHL07 colonization increased proline content while reducing oxidative damage under salt stress [61]. *M. anisopliae* MetA1 seed priming enhanced proline accumulation in rice under saline conditions, accompanied by improved growth and yield [59]. *B. bassiana* BeauA1 similarly increased proline and carbohydrate levels in rice [60], and *B. bassiana* Sar-31 enhanced proline accumulation in potato [62]. These findings indicate that promoting osmolyte accumulation is a conserved EPF-mediated mechanism across plant species.

#### Antioxidant defense activation

Salt-induced oxidative stress is counteracted by EPF through enhancement of both enzymatic and non-enzymatic antioxidant systems. *M. anisopliae* LHL07 upregulated SOD, CAT, and other antioxidant enzymes in soybean [61]. *M. anisopliae* MetA1 activated CAT, POD, APX, and glutathione S-transferase (GST) in rice while also increasing phenolic and flavonoid contents [59]. *B. bassiana* BeauA1 similarly enhanced antioxidant enzyme activities and reduced oxidative damage (lower ROS and MDA) in rice [60]. In potato, *B. bassiana* Sar-31 activated antioxidant enzymes and maintained lower MDA levels under salt stress [62]. *M. pinghaense* AAUBC-M26 also induced the production of phenolic and flavonoid compounds in tomato, contributing to oxidative stress mitigation [63]. This consistent activation of antioxidant defenses across diverse EPF-plant combinations underscores its centrality in salt stress alleviation.

#### Phytohormone modulation and growth promotion under stress

EPF endophytes enhance plant salt tolerance not only by mitigating stress but also by actively promoting growth through hormone modulation. A key mechanism is the maintenance or enhancement of auxin (IAA) production under saline conditions. *M. pinghaense* AAUBC-M26 sustained significant IAA production even at elevated NaCl concentrations (up to 200 mM in vitro), which likely contributed to maintained root growth and nutrient acquisition under stress [63]. This strain also exhibited ACC deaminase activity, pointing to potential ethylene modulation, although direct evidence for stress ethylene reduction in planta is still lacking [63]. EPF also modulates stress-related hormones to shift plants away from defensive, growth-limiting responses. In soybean, *M. anisopliae* LHL07 colonization decreased ABA accumulation while increasing jasmonic acid (JA) content under salt stress, suggesting a hormonal rebalancing that favours growth over stress-induced senescence [61].

These hormonal changes were accompanied by enhanced growth under saline conditions. In rice, *M. anisopliae* MetA1 improved shoot length, biomass, chlorophyll content, and photosynthetic efficiency under salt stress [59]. *B. bassiana* BeauA1 similarly enhanced rice growth parameters, including leaf area, leaf succulence, and relative water content [60]. In potato, *B. bassiana* Sar-31 promoted stolon production, a key agronomic trait, despite salt stress [62]. In tomato, *M. pinghaense* AAUBC-M26 improved growth under both nursery and pot culture conditions at elevated salt levels [63]. This growth promotion likely results from a combination of IAA production, nutrient mobilization, improved water relations, and stress mitigation working in concert.

### Heavy metal stress

Heavy metal pollutants (HMs), released through anthropogenic activities such as mining, sewage irrigation, and industrial emissions, represent a major category of global contaminants. These metals pose significant threats to both terrestrial and aquatic ecosystems [64, 65]. Conventionally defined by a density exceeding 5 g/cm^3^, the classification of heavy metals lacks a universally accepted standard [66]. Bioremediation is a cost-effective and environmentally sustainable technique that leverages microorganisms and plants, including hyperaccumulators and high-biomass plants [67], to mitigate or eliminate heavy metal pollutants in the soil. Recently, growing evidence has indicated that EPF function dually as both “detoxification engines” and “ecological regulators” in the remediation of heavy metal pollution (Table 3) [73].

**Table 3:** Mechanisms of heavy metal remediation by entomopathogenic fungi

Mechanism Category	Specific Mechanism/Action	Observed Effect/Outcome	Key Examples/Components Involved
Direct Mechanisms (Fungal Detoxification)	Biosorption & Immobilization	HM ions are bound to the fungal cell wall, reducing their bioavailability	Surface functional groups (e.g., carboxyl, phosphate) [68, 69]; Chitin adsorption [70]
	Bioaccumulation & Intracellular Detoxification	HMs are taken up and neutralised inside fungal cells	Internalisation via calcium channels; Detoxification by glutathione and cytochrome P450 [70]
	Biomineralization & Transformation	Secreted compounds transform HMs into less toxic or volatile forms	Secretion of organic acids (e.g., oxalic acid) to dissolve minerals [71]; MMD enzyme demethylates methylmercury [72]
Indirect Mechanisms (Plant & Rhizosphere Regulation)	Upregulation of Plant Detoxification Genes	Enhances the plant’s own ability to efflux and sequester HMs	Upregulation of Plant Cadmium Resistance (PCR) genes [73]
	Inhibition of Plant HM Uptake	Reduces the expression of root transporters that absorb HMs	Downregulation of metal transporter genes (e.g., OsNramp5) [74]
	Enhancement of Antioxidant Defences	Boosts the plant’s enzymatic and non-enzymatic systems to counteract oxidative stress	Increased activity of catalase, peroxidase, and glutathione levels [73]
	Phytohormone production and regulation	Synthesis and modulation of plant hormones	Direct hormone production: IAA [75]; Enzymatic regulation: ACC deaminase [75]; Induced plant hormone synthesis: IAA, GA, BL [74]; Hormone signaling pathways: [76]
	Reshaping the microbial community	Enriches beneficial microbial communities that support plant health	Enrichment of plant-growth-promoting endophytes [75, 76]
	HM Stabilisation in Soil	Converts HMs in the soil into stable, less bioavailable forms	Immobilisation of HMs into residual fractions (F5) [74, 75]

#### Direct mechanisms: fungal detoxification

EPF employ several strategies to tolerate, immobilize, or transform heavy metals. These include surface binding, intracellular uptake and detoxification, and biomineralization or transformation of metals into less toxic forms.

*Biosorption and immobilization.* Many EPF, especially species of *Beauveria* and *Metarhizium*, can bind metal ions to their cell surfaces. Functional groups such as carboxyl, phosphate, hydroxyl, and amino groups on the cell wall act as binding sites [68, 77, 78]. *B. bassiana* tolerates mixtures of metals such as zinc, copper, and cadmium and uses these surface groups to immobilize metal ions through biosorption. In tests with wastewater containing multiple metals, it removed up to 84.5% of the total metals present [77]. Both *B. bassiana* and *M. anisopliae* show strong biosorption capacity for cadmium and lead; *B. bassiana* performs particularly well, taking up 83.33 mg of lead and 46.27 mg of cadmium per gram of biomass [68]. Another strain, *B. bassiana* JB15, tolerates up to 5.8 mM lead and 1.78 mM cadmium, removing 52.27% and 62.38% of these metals under optimal conditions [79]. Fourier transform infrared spectroscopy studies confirm that hydroxyl, carbonyl, amino, and phosphoryl groups are involved in binding metals such as cadmium, cobalt, lead, and tin, with changes in these groups reflecting their role in metal sequestration [78]. For cadmium, *B. bassiana* Z1 achieves 71.2% removal even at high cadmium levels (10 mM), with class V chitin on the cell wall playing a key role in adsorption [70].

*Bioaccumulation and intracellular detoxification.* Metals can enter fungal cells, where they are dealt with by internal detoxification systems. In *B. bassiana* Z1, cadmium enters cells through calcium channels, with calcium/proton exchanger genes (*chaA*, *CAX*) significantly upregulated [70]. Once inside, it is detoxified by binding to glutathione, forming Cd-GSH complexes. Genes involved in glutathione synthesis (*GCLC*, *GSS*) are upregulated, while *glutathione S-transferase* genes help conjugate glutathione to cadmium [70]. Cytochrome P450 enzymes are also significantly upregulated and actively participate in detoxification; adding a cytochrome P450 inhibitor reduced cadmium removal by 45% [70]. The metal is then stored in vacuoles or pumped out of the cell by ABC transporters [70].

*Biomineralization and transformation.* Some EPF go beyond surface binding and actively transform metals into insoluble minerals or volatile forms. *B. caledonica* dissolves and transforms toxic metal minerals (including cadmium, copper, lead, and zinc) by releasing large amounts of oxalic acid [71]. The oxalate ions then react with metal ions to form insoluble metal oxalate crystals, such as cadmium oxalate, copper oxalate hydrate (moolooite), and lead oxalate, which precipitate out of solution, effectively locking the metals away in a less bioavailable form [71]. A particularly striking example of fungal adaptation is how some *Metarhizium* species gained the ability to resist mercury. Phylogenetic studies suggest that these fungi acquired a gene called Mmd (encoding methylmercury demethylase) from bacteria through horizontal gene transfer [72, 80, 81]. Together with a mercury ion reductase (Mir), *M. robertsii* converts highly toxic methylmercury into volatile elemental mercury, which evaporates from the rhizosphere and reduces the amount of mercury that plants can take up [72, 82]. Overexpressing these genes further improved mercury removal from both soil and water [82].

#### Indirect mechanisms: plant and rhizosphere regulation

EPF that tolerate heavy metals well can form close relationships with plants and help them cope with metal stress through several indirect pathways. These mechanisms involve altering plant gene expression, boosting plant defenses, producing growth-promoting substances, and reshaping the root environment.

*Upregulation of plant detoxification genes.* EPF can boost the plant's own detoxification systems. When *M. robertsii* colonizes *Arabidopsis* or rice, it reduces cadmium accumulation in plant tissues (by 21.8–24% in *Arabidopsis*) by turning on plant cadmium resistance genes (*PCR3*, *PCR9*, and *PCR12*) in *Arabidopsis* and *OsPCR1* in rice, which help pump cadmium out of cells [73]. Genes encoding cadmium-binding proteins (such as *HIPP02*, *HIPP10*, and *HIPP45*) are also upregulated, helping to sequester cadmium within plant cells [73].

*Inhibition of plant heavy metal uptake.* EPF can limit how much metal plants take up by influencing the expression of metal transporter genes. In rice, *M. robertsii* suppressed the expression of a key cadmium uptake gene (*OsNramp5*) in roots, cutting cadmium levels by up to 44.3% in roots and 24.7% in grains [74]. EPF can also help plants cope with mixtures of pollutants. For example, when rice was exposed to both lead (Pb) and nanoplastics (NP), the nanoplastics acted as carriers that increased Pb entry into roots—a "Trojan Horse" effect. Seed treatment with *M. anisopliae* SM021 disrupted this synergy, reducing Pb uptake and translocation. The soil-to-root transfer factor dropped by 35.7% under Pb stress alone and by 34.4% under combined Pb + NP stress, showing that the fungus can intercept pollutant complexes before they reach the root [76].

*Enhancement of antioxidant defenses.* Heavy metals trigger oxidative stress in plants by generating reactive oxygen species (ROS). EPF help by strengthening the plant's antioxidant systems. *M. robertsii* colonization increased the activity of key antioxidant enzymes such as catalase and peroxidase and boosted the levels of glutathione and ascorbic acid in both *Arabidopsis* and rice under cadmium stress [73, 74]. Similarly, *B. bassiana* FE14 increased superoxide dismutase and peroxidase activity in *Miscanthus floridulus* grown in cadmium-contaminated soil, helping the plants cope with metal-induced oxidative damage [75]. Proline levels also increased, contributing to osmotic adjustment and stress protection [73]. In rice under Pb and NP stress, *M. anisopliae* SM021 helped restore the antioxidant balance. While the pollutants triggered a strong oxidative burst and raised ROS levels, fungal treatment brought SOD, POD, and CAT activities back to near-normal levels and reduced lipid peroxidation. This recovery was linked to reduced expression of ROS-generating enzymes and improved metal homeostasis, shifting the plant from a state of emergency defense to one of controlled stress management [76].

*Phytohormone production and regulation.* EPF enhance plant hormone levels through two complementary mechanisms: direct production of exogenous hormones and indirect modulation of plant hormone synthesis. For direct production, *B. bassiana* FE14 synthesizes and secretes indole acetic acid (IAA), as confirmed by both colorimetric assays and HPLC–MS analysis [75]. This fungal-derived IAA directly stimulates root growth and improves nutrient and water uptake in host plants [75]. Some EPF also produce ACC deaminase, an enzyme that breaks down ACC, the immediate precursor of ethylene. By lowering ethylene levels in stressed plants, this enzyme prevents ethylene-induced growth inhibition and helps plants remain productive under metal stress [75]. For indirect regulation, *M. robertsii* colonization significantly increases the endogenous levels of IAA, gibberellins (GA), and brassinolide in rice under cadmium stress [74]. Similarly, under combined lead and nanoplastic stress, *M. anisopliae* SM021 upregulated genes involved in hormone signaling pathways, helping to restore normal growth [76]. Together, these hormone-mediated mechanisms promote overall plant vigor, enhance growth and help plants maintain productivity even under cadmium stress [74].

*Reshaping the microbial community.* EPF can change the community of microbes living in and around plant roots. When *B. bassiana* FE14 was added to cadmium-contaminated soil, it enriched beneficial bacteria in the roots, such as *Sphingomonas*, *Bradyrhizobium*, *Massilia*, and unclassified Comamonadaceae, which are known to support plant health through nitrogen fixation, nutrient uptake, and stress tolerance [75]. This shift in the root microbiome likely contributes to the overall improvement in plant performance under metal stress. Similar changes were observed with *M. anisopliae* SM021 under Pb and NP stress. Fungal inoculation boosted the abundance of beneficial genera such as *Burkholderia*-*Caballeronia*-*Paraburkholderia*, *Mucilaginibacter*, *Sphingomonas*, and *Nocardioides*, bacteria known to promote plant growth, detoxify metals, and cycle nutrients. By immobilizing Pb and trapping NPs, the fungus reduced stress on the native microbial community, allowing a more diverse and functional microbiome to rebuild around the roots [76].

*Heavy metal stabilization in soil.* EPF release organic acids and other compounds that can bind heavy metals in the soil, making them less available for plants to absorb. *B. bassiana* FE14 produces several organic acids, including oxalic, lactic, and pyruvic acid, which can complex with metals [75]. In soil tests, adding *B. bassiana* FE14 significantly reduced the amount of cadmium extracted from soil, from 26.23 mg/kg to just 5.41 mg/kg [75]. Similarly, *M. robertsii* colonization facilitated cadmium migration from exchangeable and Fe–Mn oxide-bound fractions to the more stable residual fraction in soil, further reducing cadmium bioavailability [74].

## Other abiotic stresses

Beyond drought, salinity, and heavy metal toxicity, plants face additional abiotic threats, including extreme temperatures (heat and chilling) and the emerging concern of microplastic pollution. Currently, there is no clear evidence for the role of EPF in assisting plants in resisting such stresses. However, studies have confirmed that endophytic fungi can indeed enhance their host plants’ ability to cope with these stresses. For example, the colonization of plants by beneficial fungal endophytes has been shown to confer heat stress through multiple coordinated mechanisms. These include the preservation of photosystem II quantum efficiency, optimization of water use efficiency, and maintenance of photosynthetic capacity under high-temperature stress. Additionally, endophyte-treated plants exhibit enhanced root system development, along with elevated accumulation of osmolytes and heightened activity of antioxidant enzymes, collectively contributing to heat stress resilience [83, 84]. Beyond heat tolerance, the root-associated endophyte *Serendipita indica* (formerly *Piriformospora indica*) has demonstrated efficacy in cold stress alleviation. Transcriptomic analyses revealed that *S. indica* colonization modulates the expression of cold-responsive genes associated with primary metabolic pathways, phytohormone signaling cascades, and the transport of lipids and inorganic ions, thereby reprogramming host physiology for enhanced cold tolerance [85]. The physiological functions that these fungi confer to plants are very similar to those conferred by entomopathogenic fungi, suggesting that EPF may also play a significant role in mitigating these biotic stresses. A recent study found that microplastics can significantly enrich the abundance of indigenous EPF, suggesting their potential role in degrading microplastics or mitigating microplastic pollution in plants [86]. In addition, *M. anisopliae* seed inoculation alleviated combined lead and nanoplastic toxicity in rice by reducing contaminant uptake, restoring antioxidant balance, and reshaping the rhizosphere microbiome [76]. However, whether EPF can directly mitigate the toxicity of microplastics alone and the underlying mechanisms involved remain largely unexplored and warrant further investigation. Therefore, it is imperative to conduct broader research to establish or validate the underlying mechanisms by which entomopathogenic fungi collaborate with plants to alleviate such abiotic stresses.

## Challenges and future aspects/perspectives

While EPF endophytes hold significant promise for enhancing crop resilience to abiotic stresses and advancing sustainable agriculture, several critical challenges must be addressed to unlock their full potential and achieve widespread commercial application. Furthermore, a growing body of literature urges a critical re-evaluation of the ecological risks associated with all microbial inoculants, including EPF, framing them not merely as beneficial tools but as potential “neomicrobiota” that could disrupt native ecosystems [87, 88].

The effectiveness of EPF endophytes in conferring abiotic stress tolerance is critically dependent on specific plant-fungus combinations and prevailing environmental conditions. A strain highly beneficial for one crop under a particular stress, exemplified by *B. bassiana,* which imparts remarkable resilience in tomato during drought, may prove ineffective in another crop or falter under different stresses. Systematic screening and rigorous selection of EPF strains demonstrating robust broad-spectrum stress tolerance alongside compatibility across diverse crop species and varied agro-climatic zones and soil types are therefore paramount. A profound understanding of the molecular underpinnings of this compatibility, such as intricate signalling during colonisation and the precise mechanisms for avoiding or suppressing plant defences, remains essential.

While generally beneficial as endophytes, EPF such as *Beauveria* and *Metarhizium* retain their potent entomopathogenic capabilities. An imperative need exists to ensure that strains selected for abiotic stress mitigation pose no unintended risks. A thorough and critical assessment of the pathogenicity of the endophytic EPF strain towards non-target insects, particularly vital beneficial insects such as pollinators, parasitoids, and predators, is mandatory before any field deployment. Future research should investigate whether abiotic stress tolerance traits correlate with heightened virulence or an expanded host range.

Establishing and maintaining stable endophytic colonisation of a host crop by EPF under fluctuating and often harsh field conditions, variable temperature, humidity, UV radiation, and intense competition with soil microbiota competition remains a formidable challenge. Colonisation levels achieved in controlled environments often fail to translate consistently to the field, thus significantly compromising stress tolerance efficacy. The development of robust, cost-effective inoculation methods (e.g., seed coating, root dipping, soil drench) that are meticulously optimised for diverse crops and EPF strains is crucial for ensuring reliable colonisation. Research into advanced formulation technologies, such as microencapsulation, the strategic use of adjuvants, and innovative carrier materials that protect fungal inoculants while enhancing their establishment and persistence within the host plant, is critically important.

Introducing EPF endophytes may disrupt established plant endophytic and rhizospheric microbial communities. The consequences of these interactions for overall plant health, native stress tolerance mechanisms, and the long-term efficacy of artificially introduced EPF remain poorly understood. Conducting comprehensive investigations into the ecological impact of introduced EPF endophytes on native plant microbiomes using advanced sequencing and metabolomic approaches is needed. Investigate the potential for developing synergistic microbial consortia combining EPF with other beneficial microbes (e.g., PGPR, AMF) to achieve enhanced, more resilient abiotic stress mitigation. Furthermore, the potential ecological risk of these introduced fungi becoming invasive should be considered. As with other microbial inoculants, there is a risk that non-native EPF could disrupt local microbial communities and ecosystem functions, a concern that is gaining increasing attention but remains understudied [87, 88].

While mechanisms such as antioxidant induction, phytohormone modulation, nutrient uptake enhancement, and ion homeostasis are posited (as detailed in Sections “Drought stress”-“Heavy metal stress”), the precise molecular pathways and critical fungal determinants specific (genes and metabolites responsible for conferring distinct abiotic stress tolerance traits) remain incompletely mapped. Leverage advanced molecular tools such as transcriptomics, proteomics, metabolomics, and CRISPR-Cas gene editing to meticulously dissect the underlying mechanisms of stress alleviation at the molecular level and pinpoint the essential fungal genes and metabolites involved remains very important. This fundamental knowledge is indispensable for unlocking targeted strain improvement, achieved through sophisticated genetic engineering or directed evolution, to powerfully enhance desirable traits such as osmolyte production and heavy metal chelation/efflux.

Scaling up the production of high-quality, viable, and effective EPF inoculants cost-effectively poses significant challenges. Formulations should guarantee extended shelf life, effortless application, and seamless compatibility with existing agricultural practices. It is important and paramount to optimize mass production techniques—whether solid-state or liquid fermentation and pioneer stable, user-friendly formulations specifically tailored for endophytic application. Establishing rigorous quality control standards for these inoculants is essential. In addition, navigating regulatory pathways for registering microbial products, particularly those with dual roles such as biocontrol combined with plant growth promotion or stress alleviation, can prove intricate, protracted, and highly variable across regions. Concerns about potential non-target effects and environmental persistence necessitate thorough risk assessment. Furthermore, developing science-based, streamlined regulatory frameworks specifically designed for beneficial microbial inoculants, including EPF endophytes, is crucial. Generating comprehensive safety and efficacy data packages robust enough to support registration is vital. Finally, we must actively foster productive dialogue among researchers, industry stakeholders, and regulators.

## Conclusion

EPF endophytes offer a highly promising, sustainable tool to bolster crop tolerance against environmental assaults. Unlocking their full potential demands deeper insights into plant-fungus signaling and stress response mechanisms, robust field application strategies, rigorous environmental safety assessments, and streamlined regulatory approval processes. Overcoming these critical challenges will empower the integration of EPF into climate-resilient agriculture, substantially reduce dependence on agrochemicals, and contribute vitally to global food security.

## Data Availability

Not applicable.
